# Tools for Evaluating the Quality of Life of the Paediatric Population with Primary Headaches—A Review of Selected Questionnaires

**DOI:** 10.3390/ijerph19106295

**Published:** 2022-05-22

**Authors:** Adrianna Rożniecka, Alina Minarowska

**Affiliations:** 1 Department of Nursing, Public Health School, University of Warmia and Mazury in Olsztyn, 10-719 Olsztyn, Poland; 2 Department of Pulmonology, Public Health School, University of Warmia and Mazury in Olsztyn, 10-719 Olsztyn, Poland; minar@mp.pl

**Keywords:** paediatric headache, quality of life questionnaire, primary headache

## Abstract

Primary headaches are a common health issue in the paediatric population. These conditions have a negative impact on the quality of life of patients at the development age in every area of their lives. The aim of this study is to list the tools used to evaluate the quality of life of the paediatric population with primary headaches and to discuss their advantages and limitations. Examining the quality of life of children and adolescents suffering from primary headaches is of particular importance. This is a consequence of a high disease incidence rate and a considerable negative impact of the ailment on the everyday life of this population. It is very important to conduct such examinations with specific and validated tools. It is significant because of the particular features of the areas of patients’ lives at the developmental age. Each of the available questionnaires has specific characteristics, advantages and limitations. The data accumulated in this literature review can be of help in designing research on the quality of life of children and adolescents suffering from primary headaches.

## 1. Introduction

Headache is one of the most frequently reported ailments in children and adolescents relative to the whole population [1,2,3,4]. According to some epidemiological data reviews, the mean incidence rate for unspecified headaches can be as high as 60% [2,3,4,5,6]. Tension-type headache is the most frequently reported type. Its incidence ranges from 20% to 25% in the developmental age population suffering from primary headaches. This is followed by migraines, with an incidence of 8%, according to epidemiological data [1,5]. According to the data, primary headache is often reported at the age of puberty and in childhood, with its incidence increasing with the child’s age [1,7,8].

A headache can have a considerable impact on all aspects of children’s lives and can cause emotional issues and impair their social functions. Additionally, headaches disrupt the everyday lives of children and adolescents, increasing school absenteeism and resulting in the secondary deterioration of school results [1,2]. Pain of a different nature is accompanied by other bothersome symptoms, e.g., nausea, vomiting, hypersensitivity to sound and light or vision disorders. These data lead one to the conclusion that recurring primary headaches can have a negative impact on the quality of life of children and adolescents [4,8,9].

A holistic approach to the patient must be applied in the examination of the quality of life of children and adolescents suffering from recurring primary headaches. The type and intensity of the ailment should be determined, as well as the extent of their negative impact on functioning in many areas of life [10]. The aim of this procedure is to identify factors with a particularly negative impact on patients’ well-being, which will result in developing methods for their reduction or elimination. These actions will ultimately result in the secondary improvement of the quality of life of children and adolescents with such ailments [10,11]. This can be achieved with appropriate tools. They can be used specifically and holistically to gather information on the type and nature of patients’ ailments and their health status and well-being. They can also be employed to analyse the patients’ quality of life in detail [11,12].

## 2. Primary Headache—Classification and Epidemiology

There are over 280 types of headaches in the current classification developed by the International Headache Society (IHS). This classification introduces uniform nomenclature and criteria for their identification. The ICHD 3-beta (the International Classification of Headache, 3rd edition (beta version)) classification is the third edition of the classification proposed by IHS. It is divided into four parts, which describe: first—primary headaches; second—secondary headaches; third—painful neuropathies of cranial nerves, other facial pain and other headaches; and fourth—appendix [3,6,13,14,15].

Primary headaches denote ailments in which the headache is the basic disease symptom and its essence. It is a group of chronic conditions with a complex and not fully identified etiopathogenesis [3,5,15,16]. Primary headaches include migraine, tension-type headaches, tripartite headaches and other primary headaches [6,9,13,15].

A uniform classification is of key importance, as the ailments can be properly listed owing to unified identification criteria. The majority of epidemiological studies and reviews are based on headache diagnoses according to the IHS criteria of the past years [3,6,9,16].

In a systematic review of population studies, Wöber-Bingöl showed the overall headache incidence rate in under 20-year-olds to be 54.4% [3,17]. In contrast, Abu-Arafeh estimated that headaches occur in 58.4% of children and adolescents [6,9]. There are several representative epidemiological studies that have investigated the prevalence of headaches at the national level. They differ in research methodology and the geographic area in which they were conducted. For example, Phillip et al. indicated that in a sample of the surveyed population of children and adolescents in Austria, 75.7% of respondents experienced a headache during the year (82.1% in girls and 67.7% in boys) [8]. The lowest percentage of those reporting headaches in the general population was noted among children under six years of age. Cassuci G. reported that the headache incidence in children aged three years ranges from 3–8%, and it reaches 19.5% in the group of 5-year-olds [9,15,18,19,20]. A rapid increase compared to the other age groups was noted among schoolchildren at the age of 7, in whom headaches were reported by over 50% of the population. This trend increased with the children’s age. The incidence of headaches in children and adolescents between the ages of 7 and 15 ranges from 57% to 82% [15,18]. The increase in the prevalence of headaches in children with age was also confirmed by the Philippe study. He indicated that it increased from 63.9% in the fifth grade to 80.4% in the 11th grade. The authors also reported that older participants in this study had a higher probability of experiencing tension-type headaches [8].

Migraine and tension-type headaches are the most common types in the general population. Their incidence in age groups varies depending on the diagnostic criteria [18,21]. According to some authors, these types of primary headaches should not be regarded as separate clinical diseases but as two aspects of one headache type [22]. As epidemiological data for tension-type headache (TTH) show, it accounts for as much as 24% of all the diagnosed cases of primary headache [9,23]. According to Abu-Arafeh et al., the overall incidence of migraines can be estimated to range from 7.7% to 9.1% of all diagnosed cases of headache, with the percentage being higher among girls (ca. 9.7%) than among boys (ca. 6.0%) [6,15,18,22]. The distribution of the migraine incidence between the two sexes varies depending on the age of the population under study. It is reported more frequently by boys in the group of 7-year-old children. This changes between the ages of 7 and 11: the incidence in this age group is similar in both sexes. In contrast, migraines occur more frequently in girls than in boys in the group of adolescents over the age of 11 [6,17,18]. The data show that the incidence of headaches increases with the age of patients at the developmental age [15,18,22].

## 3. Quality of Life of Children and Adolescents Suffering from Primary Headaches

Quality of life has now become an important issue, especially in health, medical and social sciences. Studies concerning the quality of life of adults, especially those with chronic diseases resulting in disability, began in the 1970s. Initial analyses of the quality of life concerning children and adolescents started about a decade later [24,25]. The definition of the quality of life itself has changed, which makes it difficult for researchers to identify a unified concept or methods of measurement [24]. According to many authors, the concept of QoL (quality of life) should be referred to as the definition of health proposed by the WHO (World Health Organization). According to this definition, health is not only the absence of disease but also well-being in many areas of life, including physical, mental, social and spiritual [24]. Therefore, the quality of life is a subjective feeling of well-being by a patient under examination. In its assessment, one should take into consideration its perception by an individual. An assessment of the quality of life of patients at the developmental age should take into consideration additional areas, such as independence and the ability to make decisions concerning oneself, the sense of self, relations with peers, ability to make friends and development opportunities [24,26].

Due to their high incidence, primary headaches are regarded as a serious population issue. This chronic ailment has a direct negative impact on functioning in all areas of life [27]. For example, Stępień et al. pointed out that migraine attacks had a negative impact on the everyday lives of the patients under study. According to their findings, incidents of migraines in adults caused driving problems in 58% of cases, problems with working with a computer in 57% cases, problems with reading in 45% and problems with general mobility in 69% [28].

Patients at the developmental age also experience the negative impact of pain on their functioning in many areas. Patients describe their headaches as chronic ailments, felt almost continuously or in frequently repeated episodes. It causes school absenteeism and affects their social relations with family and peers. Philipe et al. indicated in the results of their research that during the preceding 4 weeks, 15.6% of participants with headaches missed at least one whole school day because of a headache, and 41.9% of them reported at least 1 day on which they were unable to carry out other activities that they had wanted to [8]. Al-Hashel et al. indicated in a study of school students in Kuwait that 24.4% of respondents reported the frequent occurrence of concentration disorders during headaches, and 26% reported that they always had these disorders. One-third of the pupils surveyed missed 6–7 school days in 6 months because of headaches [4]. Patients also have lowered self-esteem and experience disorders in spending their leisure time [15,29,30,31,32,33]. Langeveld et al. also pointed to the negative impact of migraines on general everyday life, the feeling of happiness and well-being. Patients aged 12–18 years, examined by these authors, also mentioned increased fatigue and lower satisfaction with their lives and health compared to patients with no headache [32,34]. Wilkes et al., in a study conducted in Australia, also pointed to significant differences in the quality of life in all functional domains. Lower quality-of-life scores were reported by adolescents with headaches that occurred a few times a month. Even lower scores were reported for those with headaches that occurred most days or daily [35].

Some authors have reported that childhood headaches decrease the quality of life of children and adolescents in a manner similar to cancer or chronic arthritis [29,33]. There is a high risk of future clinical presentation of childhood headaches, and they are often accompanied by psychological and mental disorders [9,19,29].

## 4. Tools for Evaluating the Quality of Life of the Paediatric Population with Primary Headaches

Various tools are used to analyse the quality of life of patients with headaches. The latest studies highlight specific areas of the quality of life typical of patients suffering from primary headaches. This has led to the identification of the most suitable specific tools to assess the quality of life [27]. These questionnaires can be divided into general instruments and disease-specific questionnaires [27,31,36].

The tools used for the general assessment of the quality of patients’ lives make use of functional scales concerning various domains of life, e.g., physical, social, behavioural and mental. They are applied in population screening and are also used to monitor and compare changes in patients with various diseases. On the other hand, disease-specific instruments are used to examine the impact of specific ailments on the quality of life or limitations associated with a specific disease. These questionnaires can also apply to the effects of treatment of a specific disease. It is noteworthy that some authors have applied parallel instruments in both groups to provide a more comprehensive picture of the issue [27,36,37].

According to the literature, the Pediatric Migraine Disability Assessment (PedMIDAS) is one of the most frequently used questionnaires. It is an instrument used to assess the impact of migraines on the quality of life. It focuses on how often, in the last three months, headaches had an impact on a patient’s family or school life and on their leisure activities [29,33,38]. The questionnaire is based on another tool, i.e., the Migraine Disability Assessment (MIDAS), intended for adults aged 20–50 years, developed by Lipton and Stuart [12]. PedMIDAS is used for a subjective assessment of functioning disorders as perceived by a patient. In consequence, it can be used to assess the predicted outcome of the treatment applied [12,15]. The questionnaire was adapted by Hershey et al. and validated for patients between the ages of 4 and 18 [39]. In principle, it should be completed by the patient on their own. However, the authors allow for the cooperation of a parent/guardian (for small children who cannot read/write). One has to make sure that the answers are confirmed by the patient. The tool structure is short and simple: it consists of six questions about the migraine impact on school absenteeism, functioning in society and practising sports. When completing the questionnaire, the patient provides the number of days during the past three months when a headache had an impact on the patient’s everyday life. The questions provide for various degrees and extents of the impact [12,40]. The first three questions concern the impact of the ailment on school absenteeism. In the first question, the patient gives the number of days of absence from school, and in the second, they report the number of days with partial absence. In the third, the number of days when the patient was absent from 50% of lessons or less due to a headache is indicated. It is very important that the days taken into account in one question not be counted again in another. Question four focuses on the patient’s home life. The patient should provide the number of days when headaches impaired the process of doing the patient’s homework. The last two questions concern leisure activities in the patient’s peer group, including physical activity. Question five is about the number of days when the patient could not participate in leisure activities, and question six relates to the number of days when the patient could participate in 50% or less of the activities [41]. It is very important to provide the exact number of days when the patient did not function properly due to the headache. Due to the fact that the memory of unpleasant and painful events from the previous 3 months is uncertain and may lead to the incorrect number of days of indisposition being indicated, it is recommended to keep a prospective diary in the period preceding the completion of the questionnaire. This is significant because these numbers determine the interpretation of results. Adding up the number of days provided by the patient gives the score, which indicates the degree of disorder in their functioning. The degree is described as “low or none” in patients with a score of 10 or less. The degree is “mild/light” in patients with a score of 11–30 points. A “moderate” degree is assigned to a patient with a score of 31–50 points, and it is “severe” when the score is higher than 50 points [40,42,43]. The higher the score, the larger the negative impact on the patient’s quality of life [12,41,43]. The questionnaire in its original version can be downloaded from the Headache Center website. However, the association does not provide information on the available language versions or their validation.

The Quality of Life Headache in Youth (QLH-Y) questionnaire is another dedicated tool for primary headaches. It was developed at the Erasmus University in Rotterdam by Langeveld et al. It is used to evaluate the quality of life of adolescents with chronic headaches. The questionnaire is validated for patients aged 12 to 18 years. During the work on the questionnaire and its validation, the author demonstrated the negative impact of chronic primary headache on the quality of life of patients in the group under study. It manifested itself as greater stress and fatigue, mood deterioration and lower level of satisfaction with one’s life and health compared to their healthy peers [32,44]. QLH-Y evaluates the quality of life of patients in six subdomains: mental functioning, everyday functioning, physical functioning, social functioning, general satisfaction with one’s life and satisfaction with one’s health. The first four subdomains comprise sets of subscales. The mental functioning domain comprises the subscales: stress, harmony, fatigue, vitality, depression, good mood and optimism for the future. The everyday functioning domain comprises the subscales: impact of headache on everyday functioning and impact of headache on leisure activities. A subscale of symptoms other than a headache is included in the physical functioning subdomain [44,45,46]. The questionnaire comprises 71 items: 69 one-choice questions constructed on the Likert scale and two visual analogue scales (VASs) for questions about the patient’s general satisfaction with their life and health. The patient can choose one of four answers in each of the 69 questions, and a number (0, 1, 2 or 3) is assigned to each of the variants. This number is also the score, which is added up further in the questionnaire interpretation. The patient can choose one answer in questions 1–55, namely: 0—“rarely or never”; 1—“sometimes”; 2—“often”; and 3—“very often or always”. The answers in items 56–69 have the following meanings: 0—“not at all”; 1—“slightly”; 2—“rather a lot”; and 3—“very much”. The last two domains are covered with VAS scales of 100 mm. The task of the patient is to answer the question: how much is he/she satisfied with his/her life? The patient answers by putting a cross at a place on a line whose beginning denotes complete dissatisfaction and the end represents complete satisfaction. All of the questions concern the period of one week before the questionnaire is completed. The final scores are added up in subdomains and divided by the number of items in each of them, and then these scores are added, yielding the final result [44,45,46]. The original version of the questionnaire can be obtained from its authors. They do not share other language versions. However, the instrument has been translated and validated many times by other researchers, and, although not very popular, it has been used in scientific research, according to the literature. The questionnaire has been translated and validated for use in Poland by J. Fliciński, MD, PhD.

General instruments are also used for evaluating the quality of life of patients with primary headaches. The Pediatric Quality of Life Inventory TM (PedsQL) questionnaire is one of the most frequently used. This tool was developed in France by Varni et al. It is used to evaluate the general quality of life, with separate variants for adolescents with chronic headaches and for children and their parents/guardians. The part to be completed by the patient has been developed in several developmental formats. Several variants for patients in the following age groups were developed: 5–7 years, 8–12 years and 13–18 years. The parent’s part of the report also has several versions. They also vary depending on the patient’s age, i.e., small children (2–4 years), children (5–7 years), older children (8–12 years) and adolescents (13–18 years).

The small child parent’s part of the report does not have a counterpart in the patient’s questionnaires [47]. Apart from a general module, the questionnaire also has different modules constructed specifically for particular diseases, such as cerebral palsy, epilepsy, asthma and rheumatic diseases. This questionnaire can be used to compare the results with those for patients with other diseases, but also with healthy children [15,33]. The questionnaire can be used to assess the quality of life with 23 items concerning various domains. These include: various aspects of physical activities (8 items), emotional functioning (5 items), social functioning (5 items) and functioning at school (5 items). Patients choose the extent of the problem in each position, using a scale from 0 to 4: 0 for “it is never a problem”, 1 for “almost never”, 2 for “sometimes”, 3 for “often” and 4 for “it is almost always a problem”. This is simplified for children aged 2–5 years (0—“not a problem at all”; 2—“it is sometimes a problem”; and 4—“it is a big problem”). The items are additionally marked visually, with images of smiling or sad faces.

After the material is collected, the results are reversed and transformed linearly into a scale of 0–100. The higher the patient’s score, the higher the quality of life [47,48,49]. The questionnaire and its modules can be obtained free of charge by sending a request to the authors via the website. The instrument is available in many language versions. There is also a version translated into Polish and validated for Poland, and the questionnaire itself is widely used both in Poland and around the world.

## 5. Summary

Particular features of the questionnaires under discussion were identified for the comparative analysis of the tools used to evaluate the quality of life of the paediatric population with primary headaches. They were grouped into categories, as shown in Table 1 (original construction).

The “population parameters” category denotes the features of the group under study for which the questionnaire is intended. The target group is different for each instrument. The same applies to the ailments reported by the patients. Some tools can also be used to examine the parents/guardians simultaneously.

The “questionnaire construction” denotes the actual tool structure, the number of items/questions asked of the patient and the way in which it was built. It also covers issues from the period before the examination, which are dealt with in the analysis, and the number of subdomains to be analysed.

The “Availability and application” category covers features related to the ease of obtaining the form and the consent to its use, availability of language versions and additional modules that enable one to compare the findings with those obtained for patients with other ailments. This category also covers the commonness of use, i.e., the frequency of use by other researchers. Issues related to other applications of the questionnaire (for example, as a tool for evaluating the treatment outcome) were additionally dealt with.

Table 2 (original construction) shows a comparison of the questionnaires with respect to the parameters of the target population. According to the list, the PedsQL questionnaire is intended for patients aged 2 to 18 years, which gives it the largest age range. It has various versions for different age groups, e.g., separate ones for adolescents and for small children. Owing to this, one can be sure that each patient has the best chance of understanding the questionnaire and completing it accurately. There is only a parent-completed questionnaire for the smallest children (2–5 years of age). This may raise doubts as to whether the results reflect the patient’s feelings or rather the guardian’s observations. It is similar to the PedMidas questionnaire, which is intended for patients aged 4–18 years, and it does not have a module to be completed by a parent/guardian. However, the authors allow for its completion by the person with custody of the child. They emphasise the need to make sure that the selected answers fully reflect the child’s statements. The QLH-Y questionnaire is intended for adolescents between 12 and 18 years of age. It is used by older patients who can answer the questions themselves. This reduces the risk of collecting data biased by the subjective feelings of the parent/guardian. However, the parameters of the group under study are limited. This can make it difficult to make comparisons with other researchers’ findings.

These tools also vary with respect to the ailments reported by the group under examination. PedsQL is a general tool. It makes it possible to examine a control group of healthy children or to compare the findings with other diseases. However, its interpretation does not identify particular restrictions specific to patients with primary headaches. This is possible with the other two tools. The QLH-Y questionnaire is intended for patients reporting chronic headaches. It provides disease-specific results. PedMidas is intended for patients with migraines, which is a limitation for the selection of the study group and eligibility for the study.

Table 3 (original construction) shows a comparison of the tool construction. PedMidas is the questionnaire with the smallest number of items. Owing to this, its completion is not time-consuming, and it does not discourage the respondents from giving answers. However, due to the use of open-ended questions, which require giving a specific number of days, the study participants may find it difficult to complete it. The questions are detailed, and their construction may lead to mistakes resulting from wrong calculations of the number of days of indisposition (which is also pointed out by the questionnaire authors). Indication of an incorrect number of days of indisposition may also be caused by the fact that the participant did not use prospective diary cards in the period preceding filling in the questionnaire.

The QLH-Y questionnaire seems to be much more detailed in this regard. It includes 71 items on the Likert scale and the VAS scale. The data will be more detailed, but giving the answers is highly time-consuming. The respondents becoming bored or distracted may lead to their failure to answer all of the questions. Additionally, positions on the VAS scale are not clearly separated: there is only the minimal and maximal position, which may make the results analysis and interpretation difficult. PedsQL seems to be the optimum tool in this regard. It includes 23 items on the Likert scale, which are also suited to the developmental variants (e.g., variants marked visually with images of smiling or sad faces).

Each of the questionnaires applies to a different period under analysis, which is also shown in Table 3. PedMidas covers the period of three months preceding the study. Given the construction of the questions, it may lead to some difficulties. A patient may not remember the exact number of days of indisposition during the period of three months before the study. The data may be inaccurate with respect to the facts and invalidate the study result. The PedsQL tool applies to the period of one month before the study. However, since there are no questions specific to the restrictions caused by the disease, the results may be imprecise. A period of 1 week is analysed in the QLH-Y questionnaire. It is a strength of the questionnaire from the perspective of those researchers who want their results to reflect the actual condition of the patient. However, the period may be too short for researchers who want to examine the long-term effect of the ailment on the patients’ quality of life.

The most detailed analysis with respect to subdomains can be obtained with the QLH-Y questionnaire. It analyses 6 subdomains with a total of 14 subscales. They are selected specifically with respect to the ailments reported by the patients and their impact on the quality of life. It provides considerable opportunities for the analysis of results and formulating conclusions. PedsQL analyses four domains related to the general quality of life. PedMidas as a specific tool analyses the quality of life only in two domains, which may impose restrictions in the comparative analysis of the results.

Table 4 (original construction) shows a comparison of the questionnaires in terms of their availability and application. According to the literature, the PedsQL questionnaire was used the most frequently. Due to this and the existence of additional modules for patients with other diseases, it provides the greatest opportunities for the analysis and comparison of results. QLH-Y proved to be the least frequently used questionnaire, which may result in its limited use in comparing the findings with those obtained by other researchers. The PedMidas questionnaire was used quite frequently. It was also used in epidemiological studies and as a tool for evaluating the predicted treatment outcome.

Each questionnaire can be obtained free of charge. The PedsQL questionnaire is easily available: one can obtain it by sending a request via the website. The Polish language version of the general questionnaire—and its other modules—is available. The PedMidas questionnaire is also easily available and can be downloaded from the Headache Center website. The association only shares the original version in English. A translated version validated for use in Poland is unavailable. Access to the QLH-Y questionnaire is definitely the most difficult because, to receive it, one must write directly to its author. As the tool was developed in 1996, the authors’ correspondence details are not up-to-date, which makes contact with them difficult. Upon receiving a request, the author sends the original version of the questionnaire in English and guidelines for the interpretation of results. The author does not have a translated version validated for Polish conditions. However, such a version is available, because the questionnaire was translated and validated for Poland by J. Fliciński, MD, Ph.D. The Polish language version can be obtained by sending a request to the author of the translation after obtaining consent from the authors of the original version for questionnaire use.

There are some limitations to consider in the above review. First, the individual features of the questionnaires were selected by the authors from the perspective of a subjective analysis of tools. This may have resulted in the lack of attention to features of the questionnaire that are important for other researchers. Secondly, the review focused on data particularly relevant to the clinical use of questionnaires, and the analysis did not take into account their statistical features, e.g., validation methodology. However, it should be mentioned that the purpose of the review was to identify the specific features and limitations of the questionnaires so that researchers could choose the appropriate tool for the study design, not to compare their statistical characteristics.

## 6. Conclusions

Each questionnaire has been shown to have specific features. The tools differ with respect to their construction or the parameters of the population for which they are intended and validated. Their availability and commonness of use vary. The comparative analysis can provide guidance and assistance to researchers who are planning experiments. It will help to choose the optimum method for conducting it, suited to the type of data important for the researcher.

Considering the limitations of the most frequently used tools, which were compared by the authors in this review, in the future, it is necessary to focus on the issue of creating a modernised tool for testing the quality of life of children and adolescents with primary headaches. The issues requiring special analysis during the creation of a new tool should be: the possibility of comparing the results obtained in different age groups and the appropriate construction of items and their number in order to exclude most of the factors that may cause inaccurate or false results. It should also be noted that the quality of life should be assessed in many domains of functioning. In particular, it is also necessary to consider what period of time preceding the completion of the questionnaire will reduce the risk of obtaining inaccurate data. The modernised questionnaire should also be available and easy to obtain, for example, via a website, where its authors can be contacted and consent to its use.

## Figures and Tables

**Table 1:** Classification of instrument features into categories.

Category of a Set of Questionnaire Features	Features of the Tool
Population parameters	Age of population under study
	Reported ailments
	Examination of the parent/guardian
Construction of the questionnaire	Number of items
	Construction of an item Period under analysis Number of subdomains under analysis
Availability and application	Commonness of use
	Application
	Polish language version
	Other modules Availability of the original version

**Table 2:** Comparison with respect to the parameters of the target population.

Population Parameters			
Tool Features	PedMidas	QLH-Y	PedsQL
Age of population under study	4–18 years	12–18 years	2–18 years
Reported ailments	Migraine-type headaches	Chronic headaches	General tools
Examination of the parent/guardian	Not possible	Not possible	Possible

**Table 3:** Comparison of the questionnaire construction.

Construction of the Questionnaire			
Tool Features	PedMidas	QLH-Y	PedsQL
Number of items	6	71	23
Construction of an item	Open-ended questions (number of days required)	Likert scale + VAS scale	Likert scale
Period under analysis	3 months	1 week	1 month
Number of subdomains under analysis	2	6 (determined by 14 subscales)	4

**Table 4:** Comparison of the questionnaires in terms of their availability and application.

Availability and Application			
Tool Features	PedMidas	QLH-Y	PedsQL
Commonness of use	High	Low	Very high
Application	QoL assessment, epidemiological studies, assessment of treatment outcome	QoL assessment	QoL assessment, comparison of QoL with other diseases
Polish language version	None	Available	Available
Other modules	None	None	Available
Availability of the original version	High (free download from the website)	Low (free, direct contact with the questionnaire authors)	High (free, upon request sent via the website)

## Data Availability

Not applicable.
